# Supplementary material for: Resolving the full spectrum of human genome variation using Linked-Reads
Source: Genome Res. 2019 Apr;29(4):635–45. doi: 10.1101/gr.234443.118 (PMC6442396; doi:10.1101/gr.234443.118)
Supplement: Supplemental Material [file supp_gr.234443.118_Supplemental_longranger-2.2.zip › longranger-2.2.tar.bz2!/lr2.2-public/lib/stan/cmdstan/stan/doc/api/html/math_2functions_2_phi__approx_8hpp_source.html]

Stan: src/stan/math/functions/Phi\_approx.hpp Source File


|  |  |
| --- | --- |
|  | Stan  2.5.0  probability, sampling & optimization |


- Main Page
- Related Pages
- Namespaces
- Classes
- Files

- File List
- File Members

All Classes Namespaces Files Functions Variables Typedefs Enumerations Enumerator Friends Macros Pages

- src
- stan
- math
- functions

Phi\_approx.hpp

Go to the documentation of this file.

1 #ifndef STAN\_\_MATH\_\_FUNCTIONS\_\_PHI\_APPROX\_HPP

2 #define STAN\_\_MATH\_\_FUNCTIONS\_\_PHI\_APPROX\_HPP

3

4 #include <boost/math/tools/promotion.hpp>

5 #include <stan/math/functions/inv\_logit.hpp>

6

7 namespace stan {

8  namespace math {

9

21  template <typename T>

22  inline typename boost::math::tools::promote\_args<T>::type

23  Phi\_approx(T x) {

24  using std::pow;

25  return inv\_logit(0.07056 \* pow(x,3.0) + 1.5976 \* x);

26  }

27

28  }

29 }

30

31 #endif

stan::agrad::pow

fvar< T > pow(const fvar< T > &x1, const fvar< T > &x2)

**Definition:** pow.hpp:17

stan::math::inv\_logit

boost::math::tools::promote\_args< T >::type inv\_logit(const T a)

Returns the inverse logit function applied to the argument.

**Definition:** inv\_logit.hpp:52

stan::math::Phi\_approx

boost::math::tools::promote\_args< T >::type Phi\_approx(T x)

Approximation of the unit normal CDF.

**Definition:** Phi\_approx.hpp:23

inv\_logit.hpp


---

[ Stan Home Page ]

*© 2011–2014,
Stan Development Team.*
